# Low-Yellowing Phosphor-in-Glass for High-Power Chip-on-board White LEDs by Optimizing a Low-Melting Sn-P-F-O Glass Matrix

**DOI:** 10.1038/s41598-018-25680-6

**Published:** 2018-05-09

**Authors:** Hee Chang Yoon, Kouhara Yoshihiro, Heeyeon Yoo, Seung Woo Lee, Ji Hye Oh, Young Rag Do

**Affiliations:** 1 Department of Chemistry, Kookmin University, Seoul, 136-702 Republic of Korea; 2 Yamato electronic Co., Ltd., Kagoshima-Ken, 899-0401 Japan; 3 YEK GLASS Co., Ltd., Seoul, 06093 Korea

## Abstract

We introduce a low-melting-point (MP) Sn-P-F-O glass ceramic material into the phosphor-in-glass (PIG) material to realize an ‘on-chip’ chip-on-board (COB) type of phosphor-converted (pc) white light-emitting diode (WLED) with green (BaSr)_2_SiO_4_:Eu^2+^ and red (SrCa)AlSiN_3_:Eu^2+^ (SCASN) phosphors. The optimum Sn-P-F-O-based ceramic components can be sintered into the glass phase with a facile one-step heating process at 285 °C for 1 min. Specifically, these soft-fabrication conditions can be optimized to minimize the degradation of the luminescent properties of the red SCASN phosphor as well as the green silicate phosphor in PIG-based white COB-type pc-LEDs owing to the low thermal loss of the phosphors at low fabrication temperatures below 300 °C. Moreover, the constituents of the COB package, in this case the wire bonding and plastic exterior, can be preserved simultaneously from thermal damage. That is, the low sintering temperature of the glass ceramic encapsulant is a very important factor to realize excellent optical qualities of white COB LEDs. The optical performances of low-MP Sn-P-F-O-based PIG on-chip COB-type pc-WLEDs exhibit low yellowing phenomena, good luminous efficacy of 70.9–86.0 lm/W, excellent color rendering index of 94–97 with correlated color temperatures from 2700 to 10000 K, and good long-term stability.

## Introduction

**Figure 1:**
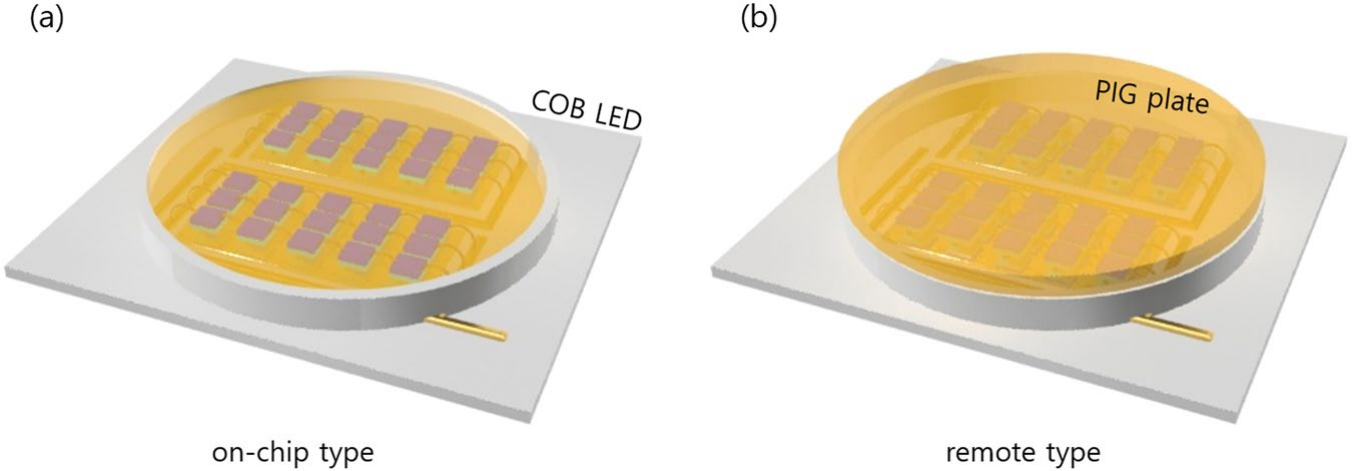
Schematic diagrams of the on-chip and remote configurations on COB-type pc-LEDs.

The use of phosphor-converted white LEDs (pc-WLEDs) and the related studies to realize inorganic color-converted phosphors have both increased rapidly over the last two decades as part of the effort to realize white color and reduce the energy consumption levels of general LED types of lighting. These pc-WLED lighting applications have prominent benefits, such as high energy efficiency levels, long lifetimes, a small size, and an economical price to attract a broad range of industrial and academic interest compared to conventional types of lighting^1–4^. However, the silicone binder used as a medium to disperse phosphors on the conventional LED package can cause serious problems, such as deterioration of the efficiency and longevity due to the yellowing phenomenon of silicone resins stemming from the highly accumulated energy of long-term heat radiation from the high-power pc-WLEDs^5–7^. During the last decade, several types of transparent glass ceramic matrixes have been developed as possible replacement candidates for silicone as a binder material to fabricate phosphor-in-glass (PIG) components that contain a certain amount of dispersed phosphor powder in a transparent glass ceramic medium (see Table 1)^8–19^. Although many papers have reported that PIGs have higher thermal conductivity levels and longer lifetimes than phosphor-in-silicone (PIS) binders, the luminous efficacy (LE) and color rendering index (CRI) of PIG-based pc-WLEDs remain inferior to those of conventional PIS-based pc-WLEDs. In order to enhance the LE and CRI of PIG-based pc-WLEDs such that they exceed the commercial levels of conformally coated red (R) and green (G) mixed phosphors on a blue LED (known as the ‘on-chip’ type), both a highly efficient phosphor configuration and a loss-free R-PIG should be carefully designed and fabricated. Thus far, the PIG architecture in pc-WLEDs prefers a remote phosphor configuration or an on-package design rather than an on-chip configuration (see Fig. 1) when manufactured with white LEDs owing to the higher fabrication temperature of PIGs, which typically exceeds 500 °C. At such a high temperature, the wire bonding of the LED package, the contact part of the electrode, and certain plastic parts are difficult to preserve after the heat treatment for the fabrication of the PIG on-chip configuration. In addition, at these fabrication temperatures, both the CRI and LE of warm white PIG-based pc-WLEDs are degraded owing to the serious problem in which the optical and crystal properties of the widely used R phosphors of (SrCa)AlSiN_3_:Eu^2+^ (SCASN) and K_2_SiF_6_:Mn^4+^, for instance, are rapidly deteriorated when fabricating R PIGs over 500 °C^20,21^.

**Table 1:** Summarization table of dual-phosphor based and/or low-temperature sintered PIG-type WLEDs in previously reported works.

Composition	Sintering temp.(°C)	Glass plate Trs.	Phosphors			Thermal conductivity (W m^−1^ K^−1^)	Thickness (*μ*m)	Luminous efficacy (lm/W)	CRI (R_a_)	CCT (K)	Operating current	Ref.
			Green	Yellow	Red							
SiO_2_-P_2_O_5_-ZnO	500–600	70%>	—	YAG	SCASN	—	~50	95.8–123.5	72.2–89.1	3290–6066	20 mA	^8^
${{\rm{SiO}}}_{2}{ \mbox{-} \mathrm{ZnO} \mbox{-} B}_{2}{{\rm{O}}}_{3}{ \mbox{-} \mathrm{Al}}_{2}{{\rm{O}}}_{{3}_{3}}{ \mbox{-} K}_{2}{\rm{O}}$	500–700	30%> (PIG)	SCGS	—	SCASN	1000	900	5.0	89.0–93.4	3391–3775	100–350 mA	^9^
SiO_2_-B_2_O_3_-ZnO-Na_2_O	500–600	60%>	—	YAG	SCASN	1.23	250	—	82–93	3789–7048	20 mA	^10^
SiO_2_-ZnO-B_2_O_3_-Al_2_O_3_-K_2_O	600	—	LuAG	—	SCASN	—	1000	5.7–8.3	80.2–90.2	3651–4258	350 mA (20 V)	^11^
SiO_2_-B_2_O_3_-ZnO-BaO-Na_2_O-K_2_O-Li_2_O-Al_2_O_3_	600	—	—	YAG	SCASN	—	90	44.1–50.4	82.6–84.5	3784–4019	500 mA	^12^
SiO_2_-B_2_O_3_-Al_2_O_3_	600	—	BMS	—	SCASN	—	500	23.1–27.2	84.2–87.2	2984–3326	500 mA	^13^
TeO_2_-Sb_2_O_3_-Li_2_O-ZnO	530	80%> (LuAG PIG)	LuAG	—	SCASN	—	~650 (Dual layer)	102.1	76.5	3410	20 mA	^14^
Sb_2_O_3_-B_2_O_3_-ZnO-K_2_O	570	—	—	YAG	YAG (Ce^3+^/Mn^2+^/Si^4+^-doped)	—	—	82–136	76.9–86.0	3582–7902	350 mA	^15^
TeO_2_-B_2_O_3_-ZnO-Na_2_O-Al_2_O_3_	570	—	—	YAG	BMA	—	400	55.1–99.2	68.4–86.0	3622–6608	350 mA	^16^
Sn-P-F-O	230	71~81%	—	YAG	—	1.259 (horizontal)	~600	108–110	60–70	3790–5415	350 mA	^17^
Sn-P-F-O	350	—	—	YAG	SCASN	—	1000	65–80	73.6–87.7	2808–5062	20 mA	^18^
Sn-P-F-O	370	85%>	—	YAG	SCASN	0.71	800	72.6–108.8	—	2662–6173	350 mA	^19^
Sn-P-F-O	285	75%>	BSSO	—	SCASN	0.712 (vertical)	~750	70.9–86.0	94–97	2817–10030	170 mA (34 V)	This work

(Abbreviations: YAG = Y_3_Al_5_O_12_:Ce^3+^, SCASN = (Sr_x_Ca_1−x_)AlSiN_3_:Eu^2+^, SCGS = (SrCa)Ga_2_S_4_:Eu^2+^, LuAG = Lu_3_Al_5_O_12_:Ce^3+^, BMS = Ba_2_MgSi_2_O_7_:Eu^2+^, BMA = BaMgAl_10−2x_O_17_:xMn^4+^, xMg^2+^, BSSO = (BaSr)_2_SiO_4_:Eu^2+^).

The authors recently developed a novel Sn-P-F-O glass material with a low melting point (MP) and found that a Y_3_Al_5_O_12_:Ce^3+^ (YAG) PIG created with it has excellent heat dissipation properties when applied to a remote PIG configuration^17^. As reported, while our low-MP glass has a sintering temperature of ~230 °C and an annealing temperature of ~150 °C, it has a low glass transition (GT) temperature (<140 °C). In this way, when a PIG with a low GT temperature is used for an on-chip configuration, the temperature close to the LED chip on the chip-on-board (COB) configuration rises to more than ~200 °C, which causes a serious problem by which the PIG on the COB LEDs can melt again and cause a loss of the emission light. Other groups have also reported low-melting-point Sn-P-F-O glass materials to reduce the sintering temperature and minimize the PL loss of the SCASN R phosphor as well as YAG yellow phosphors for the fabrication of PIGs^18,19^. However, their sintering temperatures are still higher than 350 °C, meaning that the potential for slight PL loss of R phosphors and device failures remains. It is necessary to develop PIG color converters which have MP temperatures lower than 300 °C and with improved GT temperatures which exceed 150 °C.

In this paper, to apply the PIG to the on-chip configuration of COB LEDs and to minimize the emission loss of the SCASN R phosphor-based PIG, we developed a new Sn-P-F-O-based glass material with a GT temperature higher than the ambient temperature close to the LED chip and with a sintering temperature of the PIG that does not significantly degrade the optical properties of the SCASN R phosphor or the silicate G phosphor after optimizing the optical, thermal and morphological properties of various Sn-P-F-O-based glass frits. In addition, we compare the optical properties and operational stability of on-chip PISB and PIG configurations using the newly developed Sn-P-F-O glass-based G and R phosphor-mixed PIG ((BaSr)_2_SiO_4_:Eu^2+^ (BSSO) G phosphor and SCASN R phosphor) on-chip COB-type WLEDs. We also optimize the optical performances, in this case the LEs, correlated color temperatures (CCTs), CRIs, and the special CRI for strong red (R_9_) of the G and R phosphor-mixed PIG ‘on-chip’ COB WLEDs by varying the phosphor ratio of the G and R phosphors. Furthermore, we discuss the thermal stability, high CRI and stable LEs in relation to this innovative Sn-P-F-O glass frit with a low MP and proper GT temperature for applications to PIG-contact on-chip COB WLEDs.

## Results and Discussion

Various Sn-P-F-O glass frits were synthesized to find the optimum Sn-P-F-O glass-based PIG for application to the on-chip type of COB WLEDs while comparing the degree of transparency, the morphology of the sintered glass, the MP temperature, the GT temperature of the glass frits, and the PL conversion efficiency of the PIG samples. Based on analyses of the optical and physical properties, as shown in Fig. S2 and Table S1, one optimum glass frit was selected from the various Sn-P-F-O glass frits for application to a PIG for on-chip COB WLEDs. The newly developed Sn-P-F-O glass material showed an improved GT temperature of 150 °C, a low MP temperature of 285 °C and good thermal/mechanical properties compared to those of most glass ceramic materials reported the literature (See Tables 1 and 2).

**Table 2:** Mechanical and thermal properties of the PIG plate and PISB film samples.

Sample	Hardness (Mpa)	Refractive index	Glass transition Temperature	transmittance	Transmittance After thermal aging	Density^*^ (g cm^−3^)	Specific heat^*^ (J g^−1^ K^−1^)	Thermal diffusivity^*^ (mm^2^ s^−1^)	Thermal conductivity^*^ (W m^−1^ K^−1^)
PIG plate	>1000	1.70–1.80	150–160 °C	~70%	~70%	3.635	0.469	0.418	0.712
PISB film	1–100	1.30–1.55	10–30 °C	~93%	~76%	1.254	1.363	0.277	0.474

^*^Average value for three times measurement.

**Figure 2:**
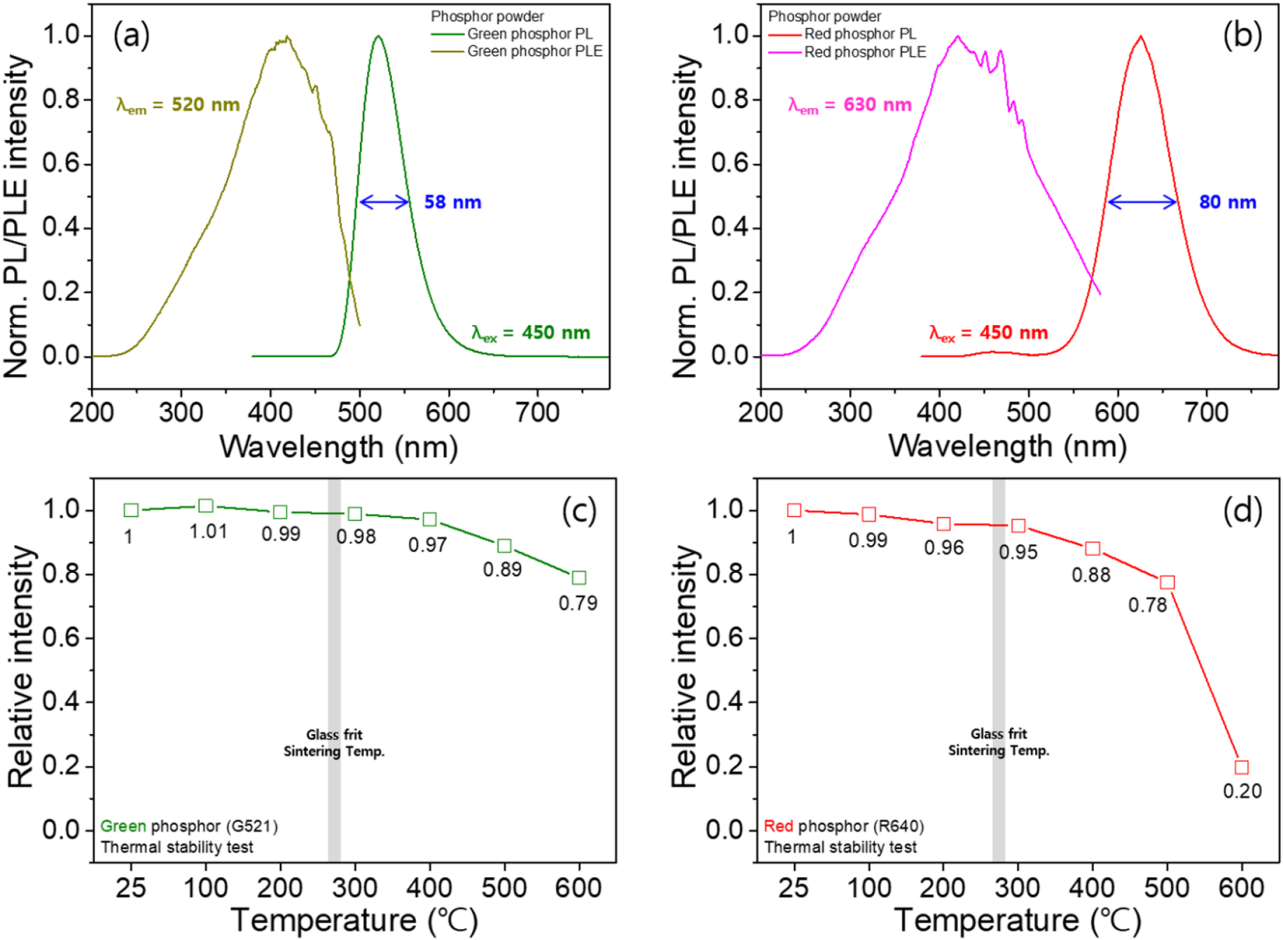
PL and PLE spectra of (**a**) green (BaSr)_2_SiO_4_:Eu^2+^ and (**b**) red (SrCa)AlSiN_3_:Eu^2+^ phosphors. Relative PL intensity of the temperature dependence of the (**c**) green (BaSr)_2_SiO_4_:Eu^2+^ and (**d**) red (SrCa)AlSiN_3_:Eu^2+^ phosphors.

The PLE and PL spectra of both the BSSO G and SCASN R phosphor powders are displayed in Fig. 2a,b. Broad PL emissions (FWHM: 58 and 80 nm) of the G and R peaks are centered at 520 nm (4f → 5d of Eu^2+^ in silicate) and 620 nm (4f → 5d of Eu^2+^ in nitride) under 450 nm excitation^22,23^. Moreover, both the G and R powder phosphors have intense PLE peaks, located in a broad range of 400–500 nm. Importantly, as the PLE ranges overlap with the blue wavelength range (~450 nm), the as-prepared BSSO G and SCASN R phosphors can be applied properly as color-by-blue convertors to realize WLEDs. Both the PL and PLE spectra of the G and R PIGs are well matched with those of the corresponding G and R powder phosphors, as shown in Fig. S3. As shown in Fig. 2c,d, the temperature dependence of the PL intensity of the G and R powder phosphors indicates that the co-sintering temperature below 300 °C reduced the PL efficiencies by only ~2% and ~5% for the BSSO G and SCASN R PIG, respectively. Based on the consideration of the upper limit (300 °C) of the co-sintering temperature, we developed Sn-P-F-O glass with a low co-sintering temperature of 285 °C. The similarity of the PLE and PL spectra between the GR phosphor powders and the PIGs confirms the negligible heat effect of the low co-sintering temperature on the PLE and PL of the GR PIGs during the co-sintering process. Compared to other glass materials in numerous previous reports, the short sintering time of our Sn-P-F-O glass materials provides another advantage with regard to the PIG production process. Our Sn-P-F-O glass materials melt quickly and can be co-sintered with silicate and nitride phosphors after co-sintering for less than 1 min. After careful optimization of the transparency level, PL intensities and external quantum efficiency (EQE) of the glass plates and the PIG plates, the sintering conditions, in this case, heating at 285 °C for 1 min, of the PIG plates were regarded as the best conditions for the fabrication of efficient G and R phosphor-mixed PIG on-chip devices and plate color converters.

**Figure 3:**
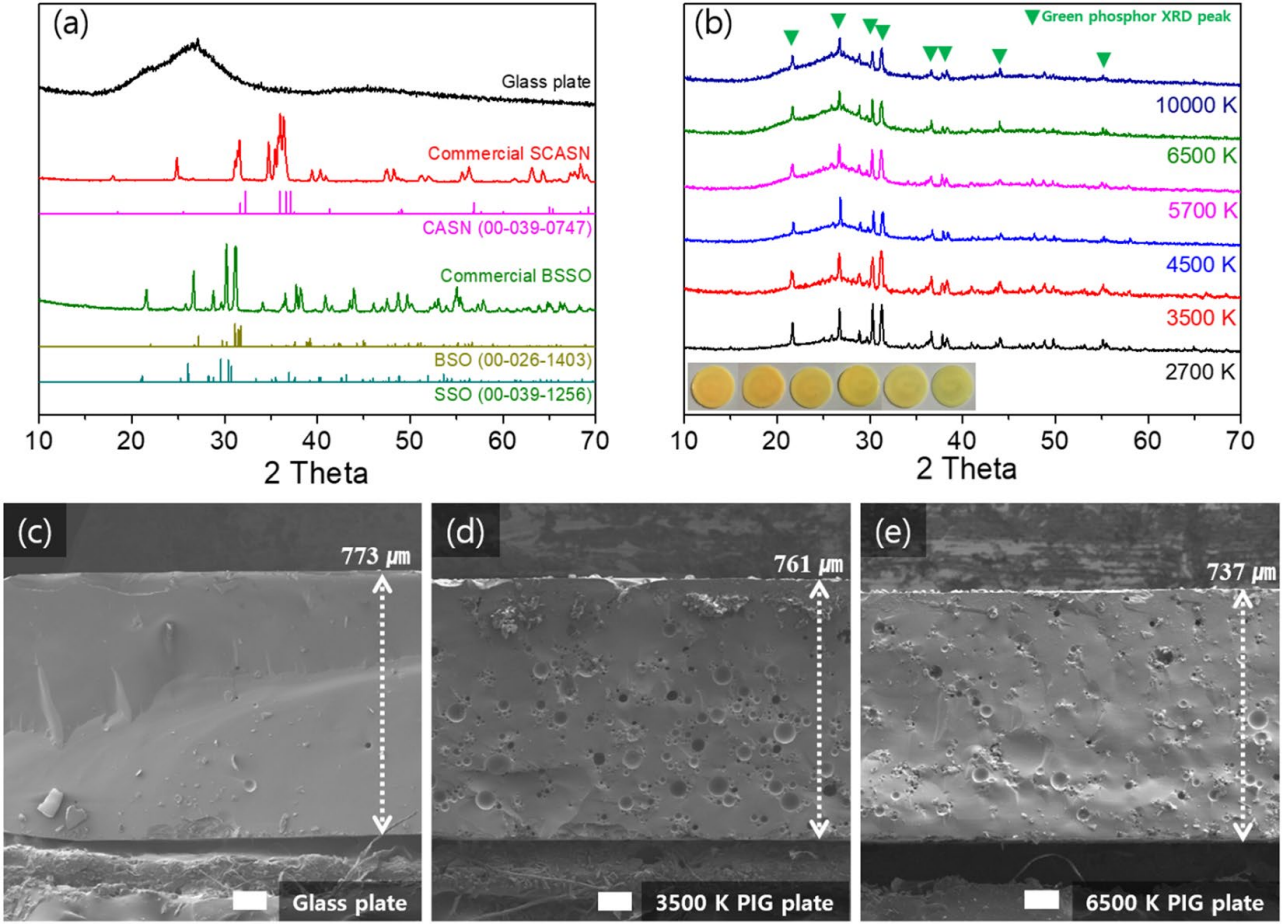
XRD patterns of (**a**) a bare glass plate, BSSO green, SCASN red powder phosphors, and (**b**) green and red phosphor-mixed PIG plates as a function of 2700–10000 K CCTs. The reference XRD peaks of BaSiO_4_, (BSO), SrSiO_4_ (SSO) (JCPDS: 00-026-1403, and 00-039-1256, respectively), and CaAlSiN_3_ (CASN) (JCPDS: 00-039-0747) are introduced for indexing the diffraction peaks of green and red phosphor, respectively. The inset shows actual images of a PIG plate in which the CCTs increase from left to right. Cross-section SEM images of (**c**) a bare glass plate, (**d**) a PIG plate at 3500 K, and (**e**) a PIG plate at 6500 K with the white bars showing a 100 μm scale.

This soft sintering condition can allow a glass plate (or a PIG plate) to be fabricated with a facile one-step heating process within 1 min. Figure 3a,b show XRD patterns of a bare glass plate^17–19^, BSSO G and SCASN R phosphor powders^22,23^, and a fabricated GR dispersed PIG as a function of the 2700–10000 K CCTs in pc-WLEDs (for comparison). In fact, the intrinsic XRD references of green (BaSr)_2_SiO_4_:Eu^2+^ (BSSO) and red (SrCa)AlSiN_3_:Eu^2+^ (SCASN) phosphors do not exist. Moreover, it is uncertain as to what the detailed ratios of Ba/Sr and Sr/Ca are in commercial green and red phosphors, respectively. Therefore, we estimated the XRD patterns of the prepared commercial green BSSO and red SCASN phosphors by comparing existing BaSiO_4_ (BSO, JCPDS: 00-026-1403) and SrSiO_4_ (SSO, JCPDS: 00-039-1256) XRD references for green BSSO and a CaAlSiN_3_ (CASN, JCPDS: 00-039-0747) XRD reference for red SCASN based on Vegard’s law. Two broad peaks of the Sn-P-F-O glass plate around 26° and 46° indicate the amorphous phase of the glass frit, and several sharp peaks of the GR PIG plates are verified as a combination of the amorphous hump of the glass matrix and the BSSO G phosphor XRD peaks while retaining the crystallinity via the co-sintering process. It is thought that the XRD peaks of the SCASN R phosphor could be concealed beneath the comparatively dominant peaks of the glass and green phosphor, as the amount of SCASN R phosphor in all of the PIG plate samples is less than 1 wt% inside the glass matrix (See Table S1).

As shown in Fig. S4, the SEM-EDX results of the BSSO G and SCASN R phosphors and the Sn-P-F-O-based glass frit show their compositional components. Figure 3c–e show cross-sectional SEM images of a bare glass plate and PIG plates containing G/R phosphors at weight ratios of the 3500 K and 6500 K WLEDs. These images clearly indicate that the phosphor particles are uniformly embedded well inside the dense glass plate with a thickness of 730–760 μm, similar in size to that used for the COB-type WLEDs. In fact, it was confirmed that the number of pores in the glass plate increased with a decrease of the CCT. This indicates that as the addition ratio of the phosphors increases, pores can be induced from the spaces created in the phosphor. These pores in PIG plates can reduce the light extraction efficiency because they induce optical scattering losses^8^. We therefore studied a sintering process involving the mixing of phosphor/glass frit and a high-viscosity solvent to carry the fluid melted glass to spaces created in the phosphor as well as a heat pressure process to reduce the number of pores. In Fig. S5, SEM-EDX and mapping analyses confirm that the BSSO G and SCASN R phosphor particles are clearly distinguished from the glass matrix. Sn- and P- rich regions are correspondingly the glass matrix and a region with a large amount of the phosphor elements, representing a region where phosphor exists. This uniform distribution of phosphor in the glass matrix can also reduce the nonuniformity of the refractive index and thus reduce the scattering loss of emission light of the PIG.

Characterization of the binding status of Sn-P-F-O glass system can be done with Fourier-transform infrared (FTIR) and X-ray photoelectron spectrum (XPS) analyses. FTIR spectroscopy was carried out to demonstrate the interlinking among the components of the Sn-P-F-O-based glass^18,24^. As shown in Fig. S6, the peak of the H-OH bending mode is indexed at 1426 cm^−1^. In addition, the bands around 1074 cm^−1^ and 514 cm^−1^ are attributed to the P-O stretching and bending modes with non-bridging oxygen, respectively. The two bands at 1010 cm^−1^ and 838 cm^−1^ indicate P-F stretching vibration. The peaks at 911 cm^−1^ and 735 cm^−1^ are prominently shown as P-O-P stretching and bending vibration, respectively, with bridging oxygen. Furthermore, the XPS spectrum of the bare glass plate was surveyed to confirm the electron binding energy in the Sn-P-F-O glass system. XPS measurements can identify the basic structural characteristics by analyzing the strongly bonded core level and the weakly bonded valence level of the amorphous glass. Figures S7 show the complete and partial compositional set of results of the XPS measurements of the glass plate. The signal peaks of Sn, P, F, and O are determined from the overall spectrum^18,25,26^. The XPS peaks of Sn 3d show two intense peaks at 486.8 eV (3d_5/2_) and 495.3 eV (3d_3/2_) in the glass matrix. The XPS peak of P 2p is located at 133.7 eV. The average spectrum of the core-level bonded O 1s is deconvoluted into non-bridging oxygen of 531.3 eV and bridging oxygen of 532.9 eV. The F 1s XPS spectrum shows two peaks in a combined shape; this is attributed to the F-Sn binding peak of 684.3 eV and the F-P binding peak of 686.9 eV^27^. These F-binding results demonstrate that both combinations play a key role in the fabrication of the low-temperature sintered glass structure.

In order for the Sn-P-F-O based glass materials to be effective when utilized as an LED encapsulant, the water resistance and thermal stability should be secured for long-term operation. To evaluate the water-environmental resistance of the Sn-P-F-O glass plate and the GR phosphor containing PIG plate, water tolerance tests were conducted by immersing the bare glass plate and the PIG plate as well as the Si binder and PISB film (See Fig. S8). The transmittances of the bare glass plate and Si binder film were maintained for three days without a loss of transparency. In the same way, the water resistance test of the PIG plate and PISB film were carried out by immersing each sample in water. The PL results of the water-immersed PIG plate and PISB tests showed that the intensity, bandwidth, and peak positions were maintained with no damage to the glass (or Si binder) or GR phosphor for three days of water immersion.

**Figure 4:**
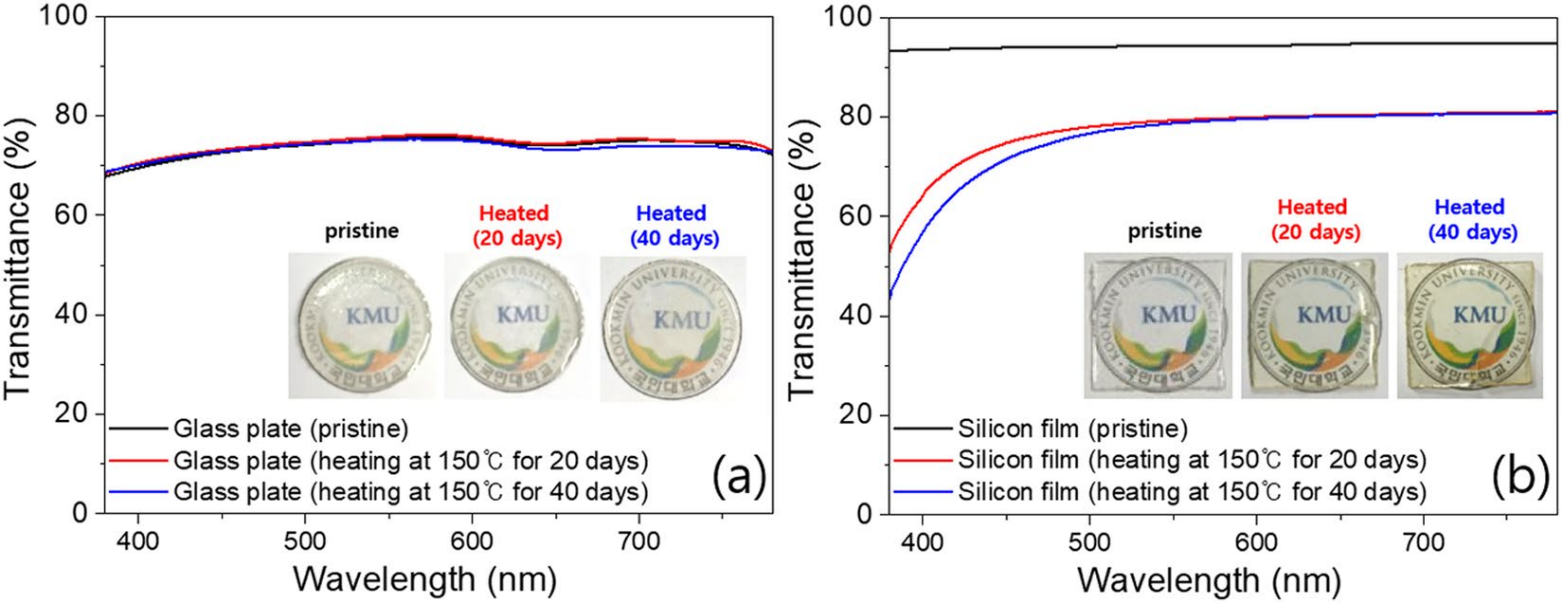
Optical transmittance spectra of (**a**) a bare glass plate and (**b**) cured silicone film in the pristine and heat-treated states.

Heat dissipation generated from the LED operation will result in poor luminous efficiency, reduced stability of the color converter, and thermal discoloration of the encapsulation binder^28^. Therefore, high thermal conductivity of the LED encapsulant is required to improve the LED package performance. In order to confirm the thermal stability, we compared the thermal properties of both the PIG plate and the PISB film by measuring transmittance (before/after heat-aging) and the thermal conductivity. Moreover, with an IR thermal camera, actual temperature images of the thermal radiation were recorded during the operation of the as-fabricated PIG and PISB on-chip COB-type pc-WLEDs under various applied currents. As is well known, silicone-binder-based LED encapsulants have a critical shortcoming in that they easily turn yellow (body color) under long-term heat radiation. To confirm the encapsulation performance of the glass plate and silicone film, the thermal stability of the Sn-P-F-O-based glass plate and traditional silicone film was investigated under 150 °C heat-aging for 20 and 40 days. Figure 4a shows that the transmittance spectra of a pristine and a heat-aging glass plate, demonstrating acceptable transmittance values that reach 68~73% in the wavelength range between 380 and 780 nm without deteriorating the transparency. A heating experiment of the silicone film was also conducted with the above-mentioned aging condition. The transmittance of the aged silicone film showed a decrease in the blue transparency of ~19.5% and 24.5% at a 450 nm wavelength after 20 and 40 day aging processes, respectively, compared to that of a pristine silicone film sample (~94% at 450 nm), with a chromaticity shift (see Fig. 4b). The transmittance loss of the G and R phosphor-mixed PIGs is attributable to the refractive index difference between the mother glass and the G and R phosphor crystals^2^, the excitation and absorption peaks of the G and R phosphors, and the scattering loss of the micro-sized phosphor powders. Compared to the refractive indices of BSSO green (~1.8) and SCASN red phosphor (~2.2), the refractive index mismatch with glass plate (1.70–1.80) is less than one of Si binder film (1.3–1.55)^29–31^. Also, all transmittance data indicate that the PIG plates with 2.01–5.90 wt% GR phosphors have acceptable transparency for pc-WLEDs. For further studies of the thermal properties of both encapsulants, we prepared a PIG plate and a PISB film samples for use in 6500 K in COB WLEDs and measured the density, specific heat, and thermal diffusivity (see Table 2). According to these factors, the thermal conductivity levels were calculated by following equation (1).

1 $$\begin{array}{rcl}{\rm{Thermal}}\,{\rm{conductivity}}\,({\rm{W}}\,{{\rm{m}}}^{-1}{{\rm{K}}}^{-1}) & = & {\rm{density}}\,({\rm{g}}\,{{\rm{cm}}}^{-3})\,\times \,{\rm{specific}}\,{\rm{heat}}\,({\rm{J}}\,{{\rm{g}}}^{-1}{{\rm{K}}}^{-1})\\  &  & \times \,{\rm{thermal}}\,{\rm{diffusivity}}\,({{\rm{mm}}}^{2}\,{{\rm{s}}}^{-1})\end{array}$$

**Figure 5:**
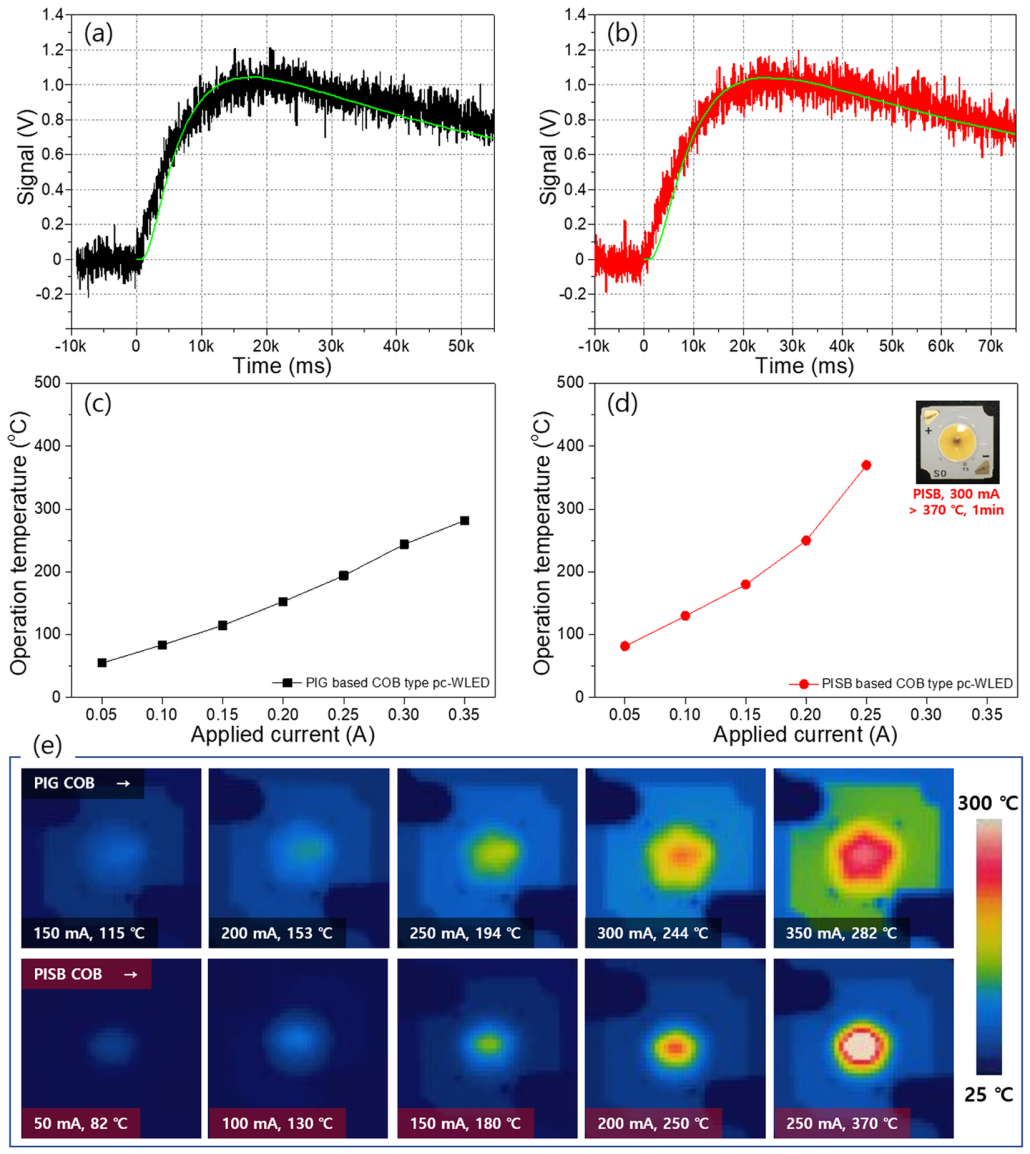
Thermal diffusivity surveys at 6500 K: (**a**) PIG plate and (**b**) PISB film with similar thicknesses of the phosphor-mixed encapsulant used on an on-chip COB LED. Operation temperatures of (**c**) the PIG-based and (**d**) the PISB-based on-chip COB WLEDs. The inset photograph shows a PISB-based COB WLED operated at 300 mA for 1 min. (**e**) Heat flux images of 6500 K PIG and the PISB-based on-chip COB WLEDs with variation of the current.

Figure 5a,b show the thermal diffusivity curve of both the GR-mixed PIG plate and the PISB film with at a thickness of approximately 750 μm, similar to the height of the color-converter/binder mounting area. This result indicates that heat flux spreading on the surface of the PIG plate occurred more quickly than that on the PISB film. That is, the thermal diffusivity of the PIG plate is higher than that of the PISB film. As a result of this calculation, the thermal conductivity of the G and R phosphor-mixed PIG is 0.72 Wm^−1^ K^−1^, which is 1.50 times higher than that of the G and R phosphor-mixed PISB. In fact, with an increase in the applied current, the actual operation temperature of the fabricated PIG/PISB-based COB-type pc-WLED (6500 K) demonstrates that the PIG causes the heat flux to shift from the point of the LED chips to the exterior plate, whereas the PISB accumulates the heat in the emissive area (see Fig. 5c–e). The better thermal and mechanical properties, the proper sintering temperature and the acceptable optical properties of the low-MP glass-based PIGs make them possible candidate as packaging materials for COB-type pc-LEDs in place of silicone as a binder material, although the optical transmittance of PIG is lower than that of the PISB sample as fabricated here. Table 2 also summarizes the mechanical properties, thermal conductivity, and optical properties of both the sintered glass plate and the silicone binder film. Consistent with results reported in our earlier publication^17^, the mechanical and thermal properties of the low-MP glass frit are superior to those of silicone binder film.

**Figure 6:**
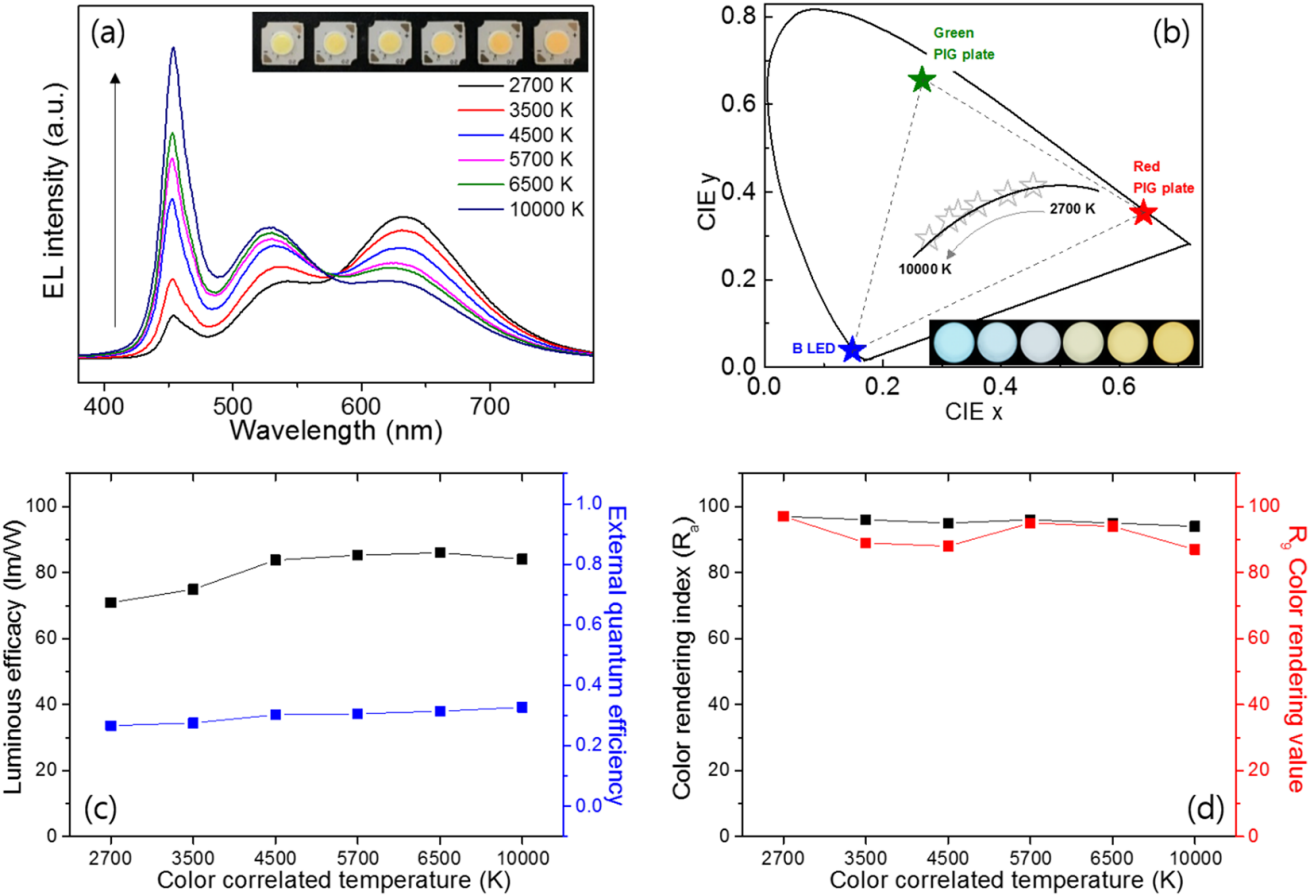
(**a**) EL spectra and (**b**) CIE color coordinates of the PIG-based on-chip COB WLEDs in the white CCT range of 2700 K and 10000 K. Insets: (**a**) actual photographs and (**b**) emission images of the fabricated PIG-based on-chip COB WLED with a decrease in the CCT from left to right. The black arrow indicates the increase in the CCT. (**c**) LE, EQE and (**d**) CRI (R_a_), and R_9_ diagrams of the PIG-based on-chip COB WLEDs.

To test the possibility of applying the G and R phosphor-mixed PIG plates to tunable pc-WLEDs in CCT ranges of 2700 to 10000 K without significantly degrading the PL properties of the R phosphors, on-chip configurations of the WLED package are constructed on the COB package, as shown Fig. 1. Here, we fabricated six different on-chip COB WLEDs by depositing the phosphor-glass frit mixture directly onto the surface of the COB package using the proper concentration ratio of and the G and R powder phosphors and low-MP glass frit, after which we co-sintered the COB LED with PIG materials at 285 °C on a hot plate (see experimental section 2.2 in detail). As mentioned in many previous reports, PIG-based remote-type pc-WLEDs appear to be preferred owing to their simple configuration given the high sintering temperature of the PIG. As previously reported, for the remote type to have LE values higher than the conventional contact type, the LED package must have an optical structure, such as specular or diffusive reflection cups, that prevents light emitted from the phosphor from being reabsorbed by the LED chips. In general, most COB LED packages do not have special optical reflector architecture owing to the complex design and the additional cost of fabricating cup-type reflectors. Therefore, if there is no optical cup-type structure in a general COB-type LED, the LEs and EQEs of PIG on-chip COB WLEDs could be higher than those of remote PIG-type COB WLEDs. The electroluminescent spectra and CIE color coordinates of the GR PIG on-chip COB package varied with the CCTs (the ratio of the G to the R phosphor) of the white LEDs, as shown in Fig. 6a,b. The blue and green intensity levels of the on-chip COB WLEDs decrease and the red intensity levels increase with a decrease of the CCTs, an increase of the total phosphor weight, and a decrease in the G/R ratio of the phosphors (see Table S1). Any white CCTs of PIG on-chip COB WLEDs can easily be obtained by the simple tuning of the G/R ratio of the phosphors and the total amounts of the phosphors, as predicted in many previous publications. The LEs and EQEs of the PIG on-chip COB WLEDs are displayed while varying the CCTs of the PIG WLEDs. Figure 6c and Table 3 indicate that the LEs of the on-chip PIG-type COB white LEDs reach moderate values between 70.9 and 86.0 lm/W, while the EQEs of the corresponding WLEDs reach 26.7–32.8%. Moreover, the results show that the CRI and R_9_ indicate very high values which exceed 94 (max. 97) and 87 (max. 97), respectively, with six different CCTs (see Fig. 6d and Table 3). The CRI and R_9_ values of artificial types of lighting are very important figures of merit to evaluate the color performance. R_9_ values of PIG COB LEDs exceeding 87 are high enough to render light of a pure red color reflected from any reddish objects. Specifically, the maximum values of the CRI of the PIG on-chip COB WLEDs were more than 96 in the CCT range of warm white at 2700 and 3500 K. These high color quality levels as well as the moderate luminous efficacy demonstrate that it is possible to fabricate warm white LEDs with on-chip PIG COB WLEDs. All COB pc-WLEDs using the on-chip PIGs with the GR phosphors in this study have excellent electro-optical energy performance values at the rated current (170 mA) and voltage (35 V) for high applied power conditions.

**Table 3:** Optical properties of PIG-based on-chip COB WLEDs.

CCT	CCT	CIE x	CIE y	CRI (R_a_)	R_9_	LE (lm/W)	LER (lm/W_opt_)	EQE
2700 K	2817	0.454	0.414	97	97	70.9	265.9	0.267
3500 K	3420	0.411	0.395	96	89	74.9	271.8	0.276
4500 K	4565	0.342	0.361	95	88	83.8	276.8	0.303
5700 K	5736	0.319	0.341	96	95	85.3	278.6	0.306
6500 K	6501	0.295	0.312	95	94	86.0	273.9	0.314
10000 K	10030	0.323	0.339	94	87	84.1	256.8	0.328

**Figure 7:**
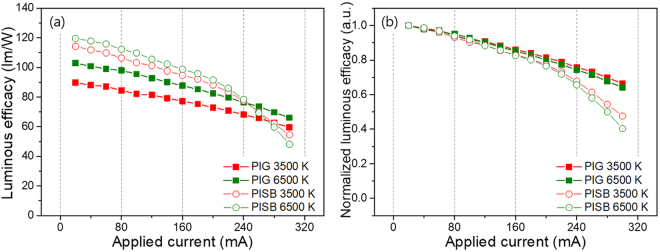
(**a**) LE (lm/W) and (**b**) normalized LE of the PIG- and PISB-based on-chip WLEDs as a function of the applied current, with a range between 20 mA and 300 mA.

**Figure 8:**
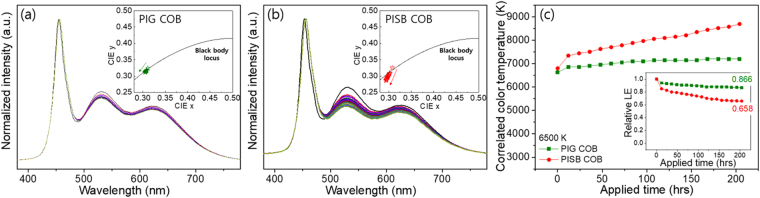
The spectrum results of the 6500 K (**a**) on-chip PIG WLED and the (**b**) on-chip PISB WLED for ~200 hours of long-term measurements. Insets show each CIE coordinate and arrows indicate an increase in the operation time. (**c**) The CCTs of the on-chip PIG and PISB WLEDs. Inset diagram shows the LE values of the corresponding on-chip PIG and PISB WLEDs.

The current dependency levels of the 3500 K and 6500 K on-chip PIG COB and on-chip PISB COB WLEDs are shown in Fig. 7a. At the rated current (170 mA), the LE values of the PISB sample at 3500 K and 6500 K were found to be 93.5 lm/W and 97.0 lm/W, respectively, approximately 15% higher than those of the PIG. This difference occurs because the transmittance of bare silicone film is 93% of the value and 18% higher than that of bare Sn-P-F-O glass plate. Figure 7b indicates that the normalized LE of both the on-chip PIG COB and the on-chip PISB COB WLEDs decreased with an increase in the applied current. In the high current region over 200 mA, the LEs of the on-chip PISB WLEDs show a dramatic drop in value compared to those of the on-chip PIG WLEDs. This explains why the measured current dependence of the PISB COB WLEDs was as high as 260 mA. According to these results, it may be that the differences in the normalized LEs between the on-chip PIG COB and the on-chip PISB COB WLEDs are due to the yellowing phenomenon of the silicone binder at the high temperature of more than 150 °C, the highly accumulated heat at the high current, and the lower thermal conductivity of the PISB films than the PIG plates. Similar to the LE dependence, the variation levels of the CIE color coordinates and the CCTs of the on-chip PIG COB are somewhat better than those of the on-chip PISB COB WLEDs, as shown in Fig. S8a~f. All data pertaining to the LE, CIE color coordinates and CCT variations of the on-chip PIG COB WLEDs collectively indicate that the on-chip PIG configuration on the COB WLEDs is more stable than the on-chip PISB COB WLEDs. Furthermore, we tested the long-term stability of the on-chip PIG COB WLED under high power COB operation. As noted above in the IR thermal image of on-chip COB WLEDs (See Fig. 5e), the operation temperature of the PISB COB WLED (~250 °C) is higher than that of the PIG COB WLED (~153 °C) at the same driving current of 200 mA. To confirm the optical properties by thermal degradation during an operation time of ~200 hrs, the on-chip PISB and PIG COB WLEDs (6500 K) were run constantly at an applied current of 200 mA with measurements taken at 12 h intervals. As shown in Fig. 8a,b, the normalized spectra of both COB WLEDs show decreases in the intensities in the green – red range, which is the emission region of the green and red phosphors, through long-term measurements. After an operation time of about 200 hrs, the LEs of the PIG and PISB COB WLEDs decreased by 13.4% and 34.2%, respectively, compared to the LEs of the initial WLED samples before the long-term measurement. Furthermore, at an identical operation time, the CCT of the PIG COB WLED changes from 6625 K to 7198 K, whereas the CCT of the PISB sample one changes from 6798 K to 8691 K (see Fig. 8c). That is, this type of optical fluctuation can be induced by thermal quenching in phosphors and structural damage to the LED chips and wire bonding components over the rated current. Moreover, these results suggest that the PIG-type encapsulant free from the yellowing problem and with high thermal conductivity is more stable than the PISB-type encapsulant in high-temperature operation conditions. Based on these optical and thermal stability data, despite the fact that the silicone binder has better transmittance than the Sn-P-F-O type of glass matrix, it is clear that the glass matrix is highly suitable for use with the on-chip type of COB WLED compared to silicone as a binder.

## Conclusion

In this study, we prepared a novel type of glass frit with a low MP temperature (<300 °C) and a moderate GT temperature (>150 °C) using Sn-P-F-O-based amorphous powders. These low-MP glass frits are promising candidates as matrix materials to replace silicone binder in phosphor-mixed remote plates and phosphor-mixed PIG on-chip types of WLEDs. The PIG plate and PIG on-chip sample were fabricated by co-sintering Sn-P-F-O glass frit mixed with BSSO G and SCASN R phosphor through a one-step heating process. XRD, FTIR, and XPS measurements confirmed the morphological structure of the obtained PIG samples and the interlinking among the glass components. We analyzed the electro-optical properties of the GR PIG plates and the GR PIG on-chip COB pc-WLEDs to assess their suitability as color-conversion composites in G and R PIG on-chip COB WLEDs. We characterized the electro-optical performance capabilities of the PIG on-chip COB WLEDs while varying various electrical parameters. These analyses indicated LE values of 70.9, 74.9, 83.8, 85.3, 86.0, and 84.1 lm/W at 2817, 3420, 4565, 5736, 6501, and 10030 K, respectively, with operation at 170 mA and 35 V. Furthermore, the warm white PIG on-chip COB WLED (2726 K) provides a CRI of 97, a R_9_ value of 97 and an EQE rate of 26.7%. The development of this glass frit with a lower co-sintering temperature can be beneficial for creating warm white color from GR PIG on-chip COB WLEDs when preventing the thermal degradation of the PL intensity of conventional phosphors, especially R phosphors. The current stability and long-term stability levels of GR PIG on-chip COB WLEDs are much better than those of GR PISB on-chip COB WLEDs under high-power conditions owing to the better thermal conductivity and low yellowing phenomenon of the PIG on-chip configurations. Therefore, the development of the low-yellowing Sn-P-F-O glass frit with a low MP temperature and a moderate GT temperature enables the fabrication of highly efficient, high-CRI, and long-term operationally stable warm white light from G and R phosphor-mixed PIG on-chip types of COB WLEDs.

## Methods

### Materials

We synthesized various MP glass frits through our previous reported protocol^17^. One optimum low-MP glass frit material was obtained a via solid-state reaction. Here, (100-x-y) mol% SnO, x mol% SnF_2_, and y mol% P_2_O_5_ powders as the glass frit precursors were prepared with composition ranges of x of 40–55 mol% and y of 20–30 mol%. These powders were ground together to make a uniform mixture and sequentially heated using a muffle furnace at 800 °C for an hour. The resulting clusters of glass frit were then placed in a cool place for thermal quenching, after which they were ground into a fine glass frit. The synthesized glass frits were filtered by sifting using a sieve to separate the variously sized powders into homogenously sized powder samples. We thus obtained glass frit about 0.1 mm in size for use in the subsequent experiment. Commercial BSSO G phosphor (SGA 521, Merck Co., Ltd.) and SCASN R phosphor (R6535, Intematix Co., Ltd.) were purchased and utilized to fabricate PIG plates for high rendition color. In addition, a silicone binder (OE-6636 A/B kit, Dow Corning) and blue COB LEDs (λ_max_ = 450 nm, Seoul Semiconductor Co., Ltd.) were used in this study.

### Fabrication of PIG plates and PIG-based on-chip COB pc-WLEDs

In Fig. S1, the entire fabrication process of the PIG plate on the blue COB LED is depicted in detail. To fabricate the on-chip type PIG-based COB WLEDs, we prepared BSSO G phosphor, SCASN R phosphor and the as-synthesized glass frit. First, the BSSO G phosphor and SCASN R phosphor were mixed at G/R phosphor weight ratios of 5.43, 6.00, 7.00, 8.20, 8.35, and 9.29 and were then added to glass frit to reach 4.5, 3.5, 2.4, 1.87, and 1.44 G/R phosphors wt% levels, for 2700, 3500, 4500, 5000, 6500, and 10000 K, respectively (see detail in Table S1). Next, amounts of 0.05 g of mechanically mixed phosphors/glass frit were loaded uniformly onto round holes 0.7 mm in height and 6 mm in diameter of blue COB LEDs and then co-sintered using a hot plate of 285 °C for 45–60 s. To obtain round-shaped PIG plate with 20 mm diameters and of the same thickness as the COB LED, 0.65 g of the as-blended phosphor/glass frit mixture was placed on a 10-mm diametric Al dish and heated in a manner identical to the above sintering condition.

### Fabrication of PISB based on-chip COB type pc-WLEDs

To fabricate the PISB-based on-chip COB-type pc-WLEDs, silicone binder OE-6636 A and B pastes were prepared as a mixture at a weight ratio of 1:2. The BSSO G phosphor and SCASN R phosphor were mixed at G/R weight ratios of 5.41 and 7.00 and were then added to the as-obtained silicone binder A/B mixture to reach G/R phosphors wt% levels of 11.3 and 8.0 at 3500 K and 6500 K, respectively (see details in Table S1). The phosphor-mixed silicone binder was moderately loaded into round holes 6 mm in diameter on blue COB LEDs and sequentially cured at 150 °C in an oven for one hour.

### Characterization

The transmittance, photoluminescence (PL) and PL excitation (PLE) spectra of the PIG and PISB samples were recorded using a Xe lamp and a spectrophotometer (Darsa, PSI Trading Co., Ltd.). Microstructural images were studied through a scanning electron microscope (SEM, JSM-7401F, JEOL Ltd.) equipped with an energy dispersive spectrometer (EDS). The crystal structures of the as-fabricated PIG samples were analyzed using X-ray diffraction (XRD) with Cu Kα radiation (D-max 2500, Rigaku) at an operating voltage of 40 kV. The density, specific heat and thermal diffusivity for calculating the thermal conductivity levels were measured using an electronic densimeter (MD-300S, Alfa Mirage), the plastics-differential scanning calorimetry method (DSC Q20, TA) and by thermal diffusivity measurements (LFA 447 NanoFlash, Netzsch), respectively. The electroluminescence spectra, CCT, luminous flux, and color rendering indices (CRIs) of the PIG-based COB WLEDs were measured in an integrated sphere using a spectrophotometer (Darsapro-5000, PSI Co., Ltd.). The Fourier transform infrared (FTIR) spectrum of the as-obtained glass plate was determined via an IR spectrophotometer (Nicolet iS50, Thermo Fisher Scientific Co. Ltd.) by an attenuated total reflection (ATR) module. The (X-ray photoelectron spectroscopy (XPS) spectra of the glass plate were measured using a monochromatic Al Kα (1486.6 eV) source operated at 12 kV and 3 mA (K-alpha, Thermo VG) with Ar ion gun sputter etching.

## Electronic supplementary material

Supplementary Information

## References

[1] Mueller-Mach R, Mueller GO, Krames MR, Trottier T (2002). High-Power Phosphor-Converted Light-Emitting Diodes Based on III-Nitrides. IEEE J. Sel. Top Quant..

[2] Zhang R (2014). A New-Generation Color Converter for High-Power White LED:Transparent Ce^3+^:YAG Phosphor-in-Glass. Laser Photonics Rev..

[3] Oh JH, Oh JR, Park HK, Sung YG, Do YR (2011). New paradigm of multi-chip white LEDs; combination of an InGaN blue LED and full down-converted phosphor-converted LEDs. Opt. Express.

[4] Chen D, Chen Y (2014). Transparent Ce^3+^:Y_3_Al_5_O_12_ Glass Ceramic for Organic-Resin-Free White-Light-Emitting Diodes. Ceram. Int..

[5] Chen D (2015). Advances in Transparent Glass-Ceramic Phosphors for White Light-Emitting Diodes-A Review. J. Eur. Ceram. Soc..

[6] Zhang XJ (2014). Highly Thermally Stable Single-Component White-Emitting Silicate Glass for Organic-Resin-Free White-Light-Emitting Diodes. ACS Appl. Mater. Interfaces.

[7] Zhang XJ (2017). All Inorganic Light Convertor Based on Phosphor-in-Glass Engineering for Next-Generation Modular High-Brightness White LEDs/LDs. ACS Photonics.

[8] Ahn SH (2017). Phosphor-in-glass thick film formation with low sintering temperature phosphor silicate glass for robust white LED. J. Am. Ceram. Soc..

[9] Kim E (2016). Effective Heat Dissipation from Color-Converting Plates in High-Power White Light Emitting Diodes by Transparent Graphene Wrapping. ACS Nano.

[10] Han K, Lee SH, Choi YG, Im WB, Chung WJ (2016). Improved color rendering index and thermal stability of white LEDs with phosphor-in-glass using the SiO_2_-B_2_O_3_-ZnO-Na_2_O glass system. J. Non-Cryst. Solids..

[11] Kim E (2016). Facile One-Step Fabrication of 2-Layered and 4-Quadrant Type Phosphor-in-Glass Plates for White LEDs: An Insight into Angle DependentLuminescence. Opt. Mater. Express.

[12] Peng Y (2017). Facile Preparation of Patterned Phosphor-in-Glass with Excellent Luminous Properties Through Screen-Printing for High-Power White Light Emitting Diodes. J. Alloys Compd..

[13] Peng Y (2016). Luminous efficacy enhancement of ultraviolet-excited white light-emitting diodes through multilayered phosphor-in-glass. Appl. Opt..

[14] Xiang R, Liang XJ, Li PZ, Di XX, Xiang WD (2016). A thermally stable warm WLED obtained by screen-printing a red phosphor layer on the LuAG: Ce^3+^ PiG substrate. Chem. Eng. J..

[15] Chen D, Xu W, Zhou Y, Zhong J, Li S (2017). Color Tunable Dual-Phase Transparent Glass Ceramics for Warm White Light-Emitting Diodes. J. Mater. Chem. C.

[16] Wang B (2016). Non-Rare-Earth BaMgAl_10−2x_O_17_: xMn^4+^, xMg^2+^: A Narrow-Band Red Phosphor for Use as A High-Power Warm w-LED. Chem. Mater..

[17] Yoo H (2016). containing glass matrix for the fabrication of phosphor-in-glass for use in high power LEDs. RSC Adv..

[18] Chen D, Yuan S, Li X, Xu W (2017). Dual-phase phosphor-in-glass based on a Sn–P–F–O ultralow-melting glass for warm white light emitting diodes. RSC Adv..

[19] Xiang X (2017). Towards long-lifetime high-performance warm w-LEDs: Fabricating chromaticity-tunable glass ceramic using an ultra-low melting Sn-P-F-O glass. J. Eur. Ceram. Soc..

[20] Lee YK, Kim YH, Heo J, Im WB, Chung WJ (2014). Control of Chromaticity by Phosphor in Glasses with Low Temperature Sintered Silicate Glasses for LED Applications. Opt. Lett..

[21] Wang L (2015). Highly Efficient Narrow-Band Green and Red Phosphors Enabling Wider Color-Gamut Led Backlight for More Brilliant Displays. Opt. Express.

[22] Zhang X, Tang X, Zhang J, Gong M (2010). An efficient and stable green phosphor SrBaSiO_4_:Eu^2+^ for light-emitting diodes. J. Lumin..

[23] Watanabe H, Wada H, Seki K, Itou M, Kijima N (2008). Synthetic Method and Luminescence Properties of Sr_x_Ca_1−x_AlSiN_3_: Eu^2+^ Mixed Nitride Phosphors. J. Electrochem. Soc..

[24] Liu H, Ma J, Gong J, Xu J (2015). The structure and properties of SnF_2_-SnO-P_2_O_5_ glasses. J Non-Cryst Solid..

[25] Anma M, Yano T, Yasumori A, Hiroshi K, Yamane M (1991). Structure of glasses in the system Sn-Pb-P-F-O. J. Non-Cryst. Solids.

[26] York-Winegar J, Harper T, Brennan C, Oelgoetz J, Kovalskiy A (2013). Structure of SnF_2_-SnO-P_2_O_5_ glasses. Phys. Procedia.

[27] Osaka A, Miura Y (1991). Bonding State of Fluorine in Lead-Tin Oxyfluorophosphate Glasses. J. Non-Cryst. Solids.

[28] Yoon SW, Park HK, Oh JH, Do YR (2014). Full Extraction of 2D Photonic Crystal Assisted Y_3_Al_5_O_12_:Ce Ceramic Plate Phosphor for Highly Efficient White LEDs. IEEE Photonics J..

[29] Frechette VD, Andrews AI (1944). Investigation of Reaction of simple Magnesia Spinels with Alkaline Earth-ortho-silicate in Solid State. J. Amer. Ceram. Soc..

[30] Jaeger F, Van Klooster H (1918). Investigations in the field of silicate chemistry. IV. Some data on the meta- and orthosilicates of the bivalent metals: beryllium, magnesium, calcium, strontium, barium, zinc, cadmium, and manganese. Proc. K. Ned. Akad. Wet..

[31] Mikami M (2013). Computational Chemistry Approach for White LED (Oxy)Nitride Phosphors. ECS J. Solid State Sci. Technol..

