# Supplementary material for: Low-Yellowing Phosphor-in-Glass for High-Power Chip-on-board White LEDs by Optimizing a Low-Melting Sn-P-F-O Glass Matrix
Source: Sci Rep. 2018 May 9;8:7412. doi: 10.1038/s41598-018-25680-6 (PMC5943285; doi:10.1038/s41598-018-25680-6)
Supplement: Supplementary file 1 — Supplementary Information [file 41598_2018_25680_MOESM1_ESM.pdf]

SUPPORTING INFORMATION FOR:

Low-Yellowing Phosphor-in-Glass for High-Power Chip-on-board White LEDs by Optimizing a Low-Melting Sn-P-F-O Glass Matrix

Hee Chang Yoon[1], Kouhara Yoshihiro[2], Heeyeon Yoo[1], Seung Woo Lee[3], Ji Hye Oh[1], and Young Rag Do[1,*]

[1]Department of Chemistry, Kookmin University, Seoul 136-702, Republic of Korea

[2]Yamato electronic Co., Ltd., Kagoshima-Ken, 899-0401, Japan

[3]YEK GLASS Co., Ltd., Seoul, 06093, Korea

[*] E-mail address: yrdo@kookmin.ac.kr

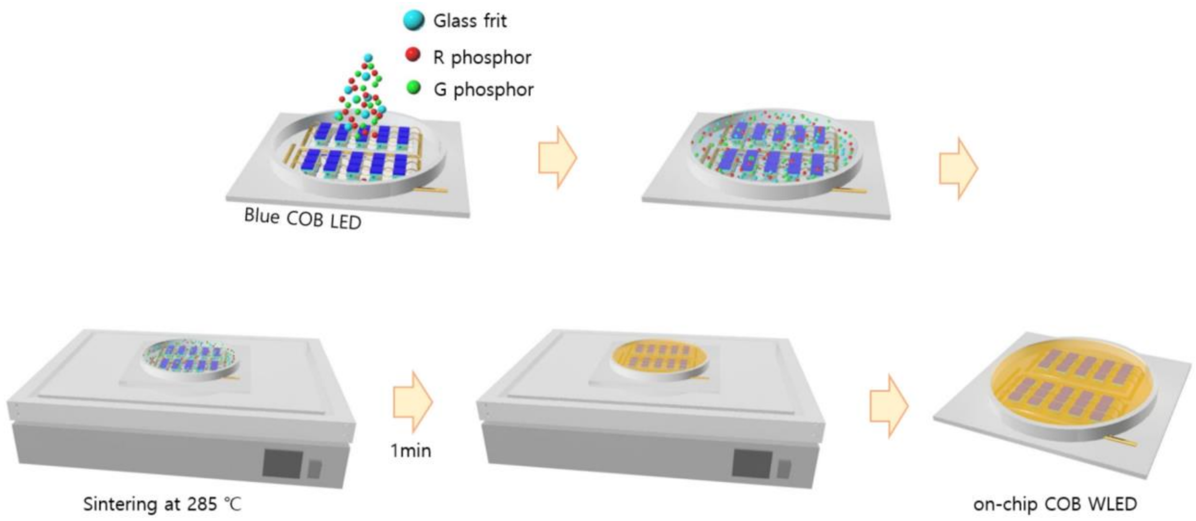

Figure S1. Schematic diagrams of the overall fabrication process of the PIG-based on-chip COB-type pc-WLED.

Table S1. The green and red phosphor weight ratio with glass frit or silicone binder according to the CCTs.

**Sample (in COB)**		**G phosphor (wt%)**	**R phosphor (wt%)**	**Total phosphor wt% in binder**	**G/R**
PIG	2700 K	4.92	0.98	5.90	5.02
	3500 K	4.35	0.82	5.17	5.30
	4500 K	3.2	0.56	3.76	5.71
	5700 K	2.35	0.33	2.68	7.12
	6500 K	2.1	0.25	2.35	8.40
	10000 K	1.81	0.2	2.01	9.05
PISB	3500 K	10.5	2.18	12.68	4.82
	6500 K	7.16	0.95	8.26	7.54

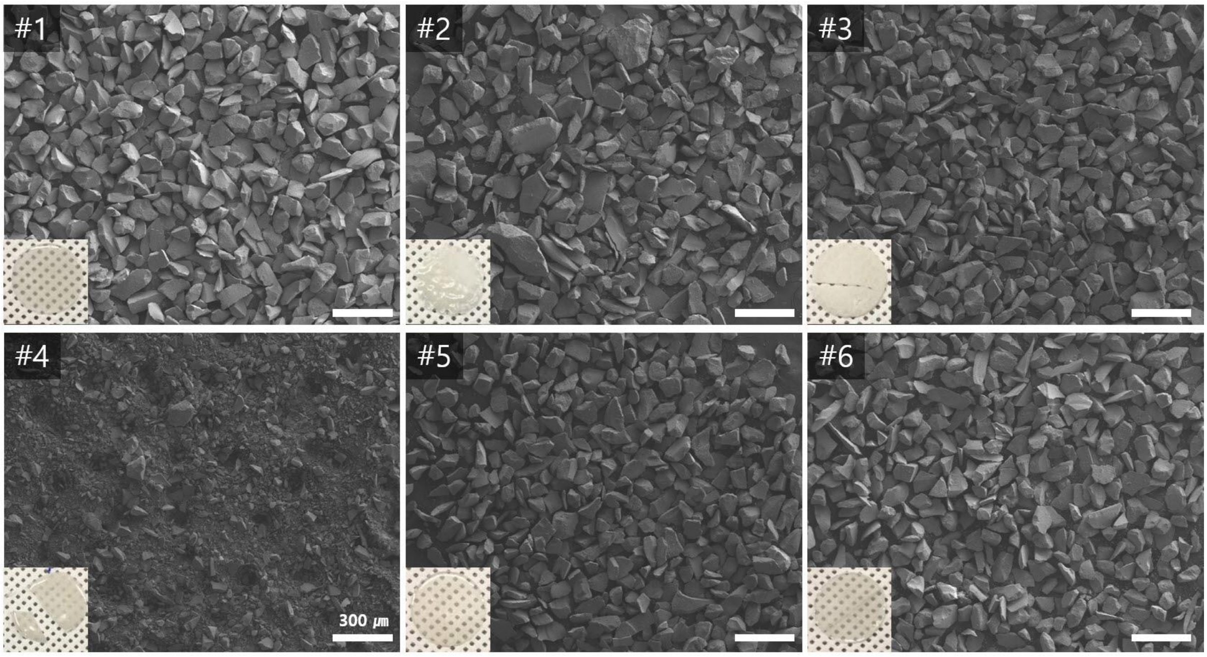

Sample #	EDX based atomic %				Trs (%)	Sintering temp. (℃)
	Sn	P	F	O		
1 (use in this work)	17.11	11.88	8.00	63.01	73.1	~ 285
2	16.98	10.97	8.70	63.34	21.8	~ 285
3	17.32	9.65	12.63	60.40	16.1	~ 285
4	20.16	9.93	13.70	56.20	58.9	~ 250
5	19.07	10.28	13.93	56.71	79.1	~ 230
6	18.77	9.06	17.00	55.16	70.4	~ 210

Figure S2. SEM images (with 300 μm scale bar) and EDX data of several types of glass frit obtained by compositional variable tests with Sn, P, F, and O elements. Moreover, summary of transmittance levels and the sintering temperatures of glass frits with compositional variables of the Sn, P, F, and O elements. Insets show each real image of sintered glass plate.

We optimized the addition ratio of glass frit precursors to fabricate transparent glass encapsulant at low temperature. Importantly, we confirmed that the ratio of F element, which is added to fabricate the glass frit, can control the glass transition temperature and low sintering temperature in obtaining the glass plate.

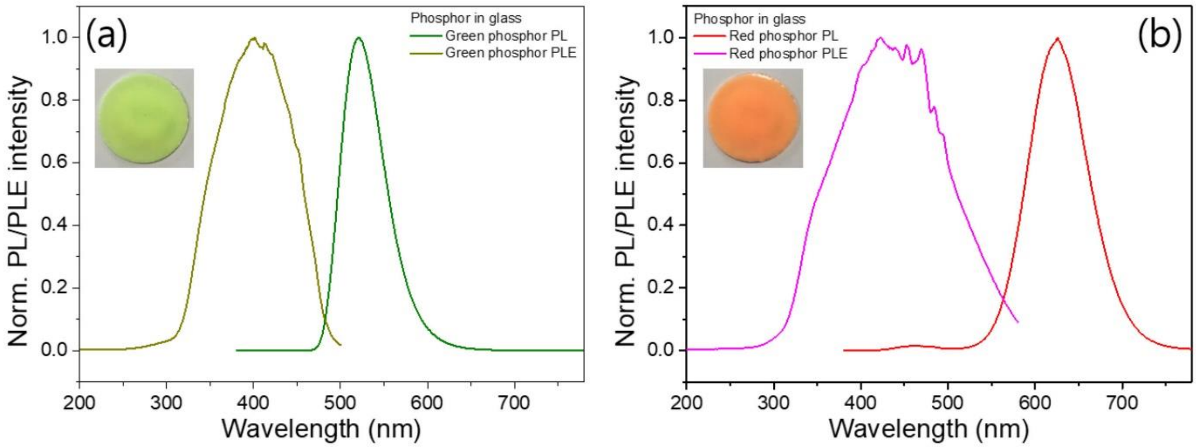

Figure S3. PL and PLE spectra of (a) a green emissive BSSO-based PIG plate and (b) a red emissive SCASN based PIG plate. Insets display actual images of each PIG plate.

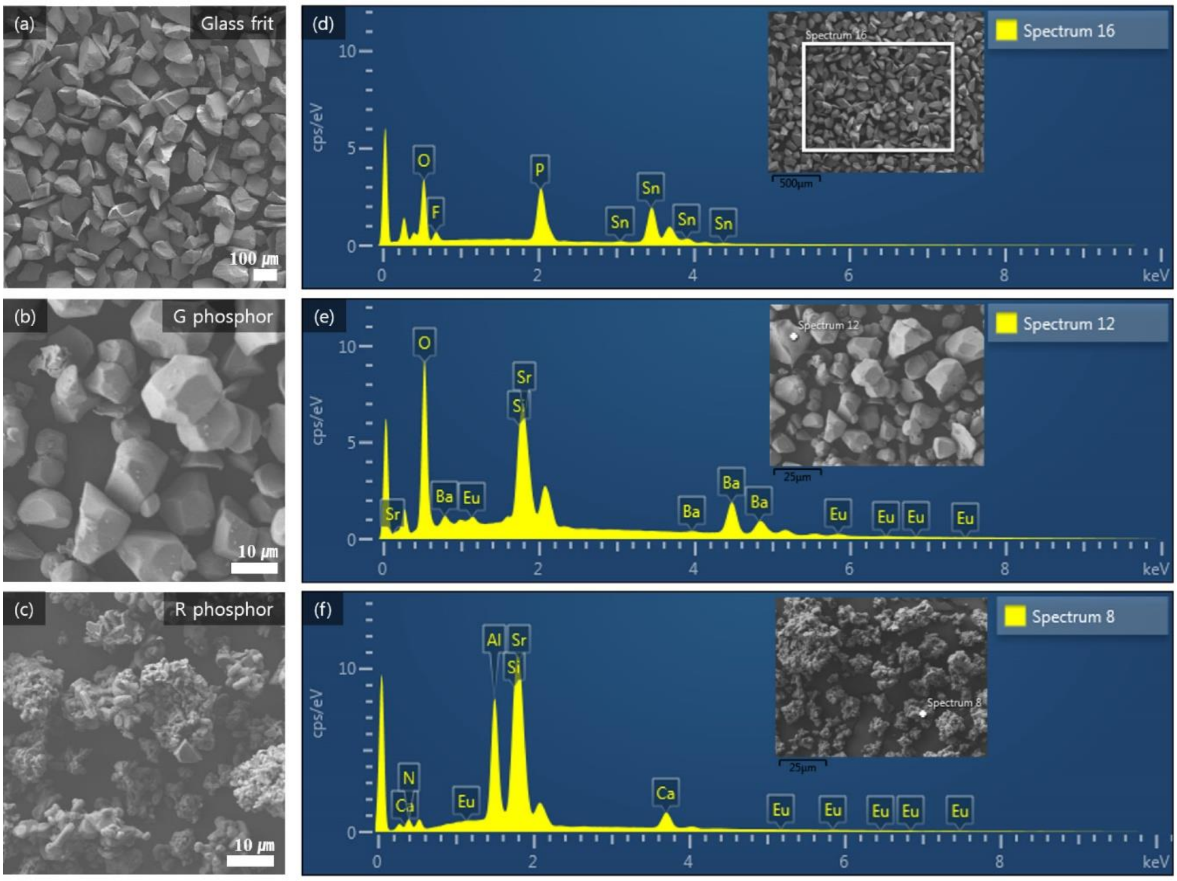

Figure S4. SEM images (a – c) and EDX spectra (d – f) of optimum glass frits, green emissive BSSO phosphor powders, and red emissive SCASN phosphor powders in the order of the figure sequence.

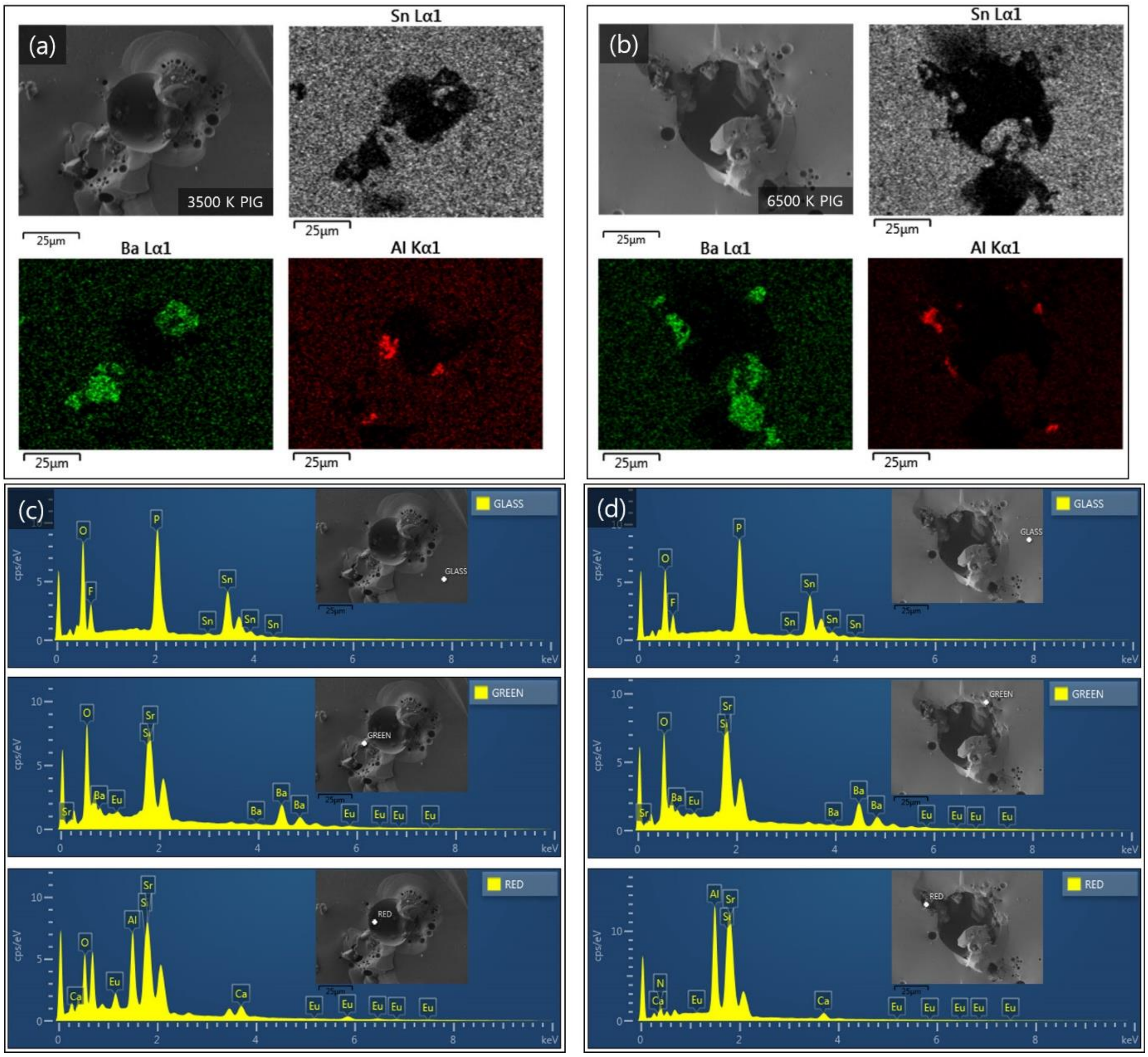

Figure S5. Here, (a) and (b) exhibit the EDX mapping results of PIG plates at 3500 K and 6500 K, respectively. The mapping colors of white, green, and red indicate the constituent elements of Sn, Ba, and Al that are present only in the glass frit, the green phosphor, and the red phosphor, respectively. Additionally, (c) and (d) show the EDX spectra of the corresponding PIG plates at 3500 K and 6500 K, respectively. These EDX images demonstrate that the compositions are different for each position analyzed in the cross-section of the PIGs.

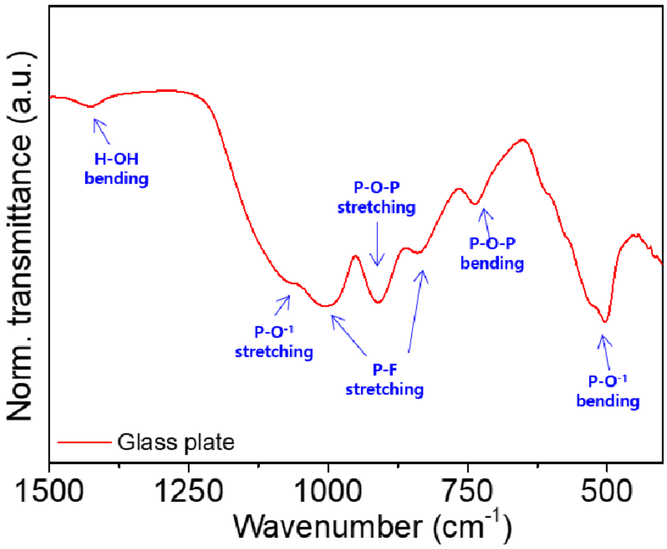

Figure S6. FTIR spectrum of a bare glass plate in the wavenumber range of 1500 to 400 cm^{-1}.

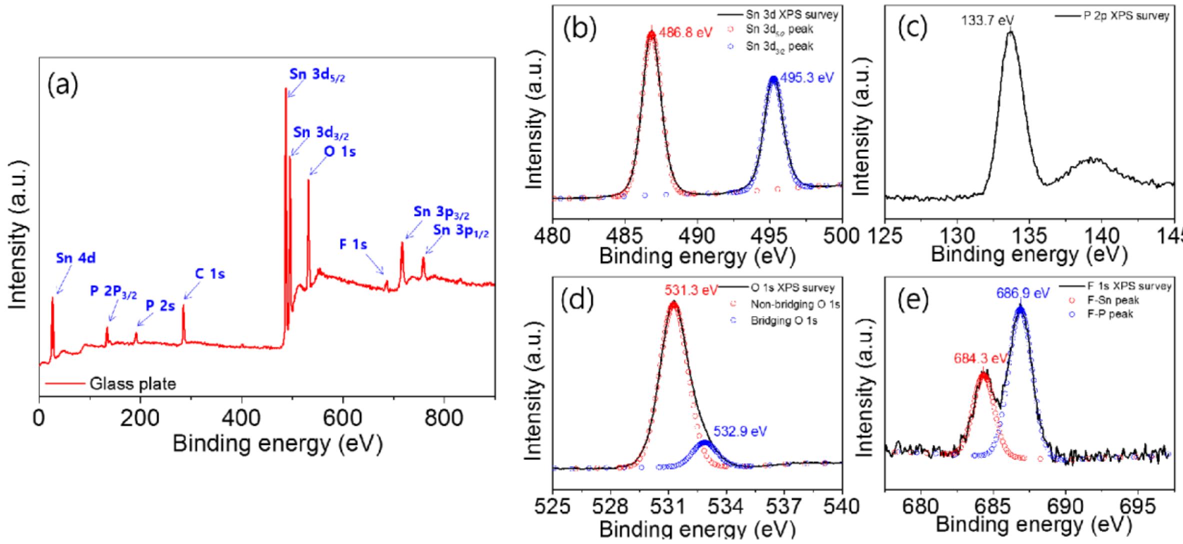

Figure S7. (a) A complete XPS survey image of a bare glass plate. Partial compositional XPS spectrum of (a) Sn, (b) P, (c) O, and (d) F bonded on a bare glass plate.

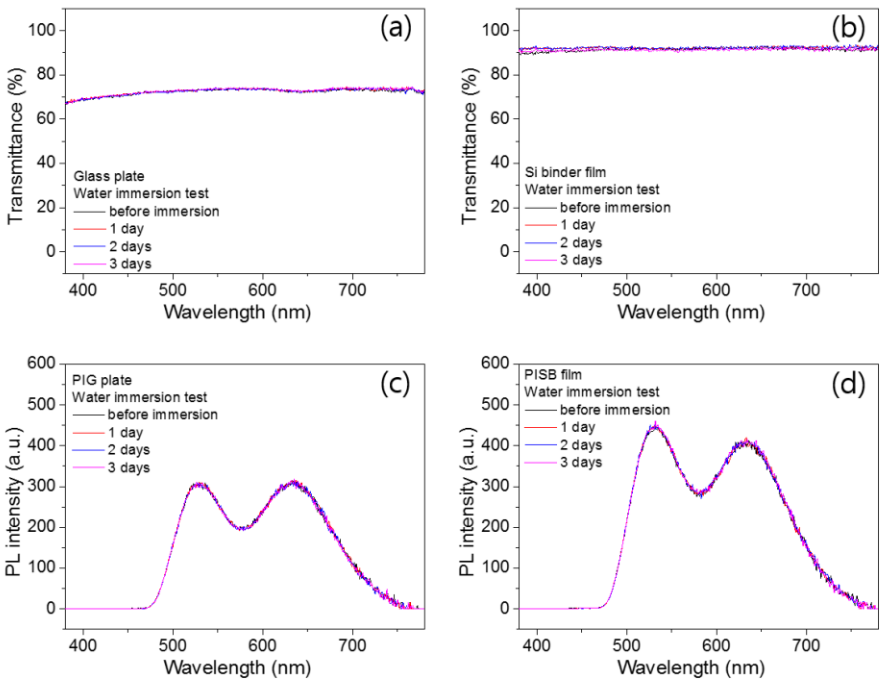

Figure S8. The transmittances of (a) glass plate and (b) Si binder film, and the PL spectra of (c) PIG plate and (d) PISB film as the resultants of water-immersion test for 3 days.

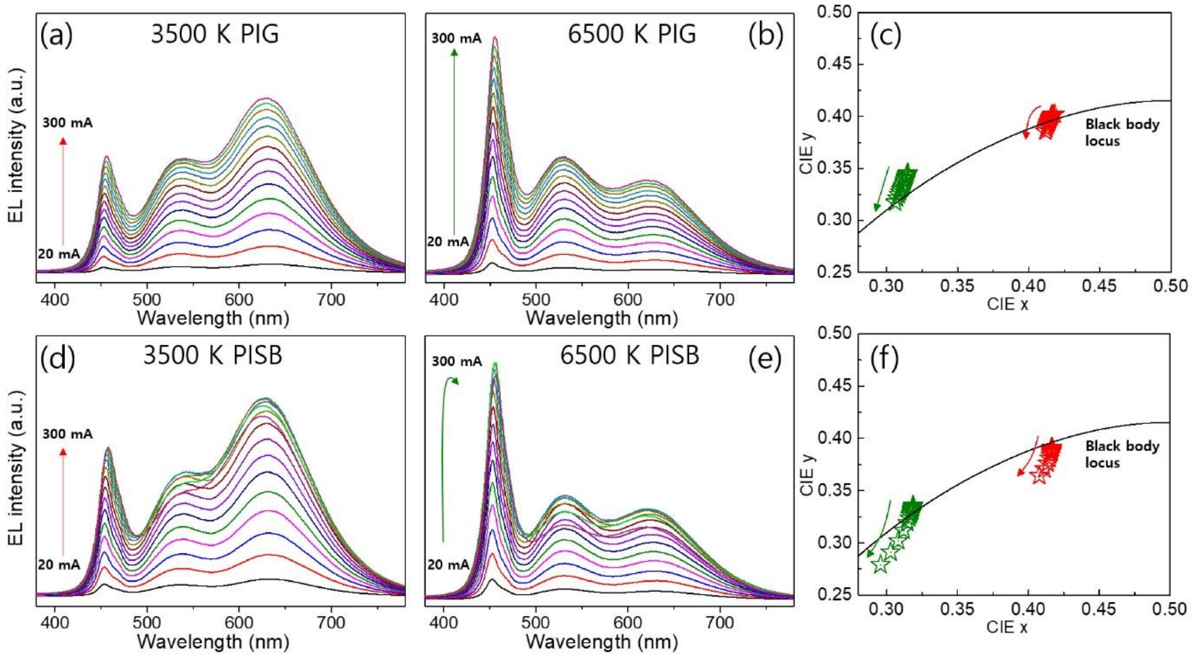

Figure S9. (a) and (b): spectra and (c) CIE color coordinates of 3500 K and 6500 K on-chip PIG-based COB WLEDs with an increase in the applied current. (d) and (e): spectra and (f) CIE color coordinates of 3500 K and 6500 K on-chip PISB-based COB WLEDs with an increase in the applied current.

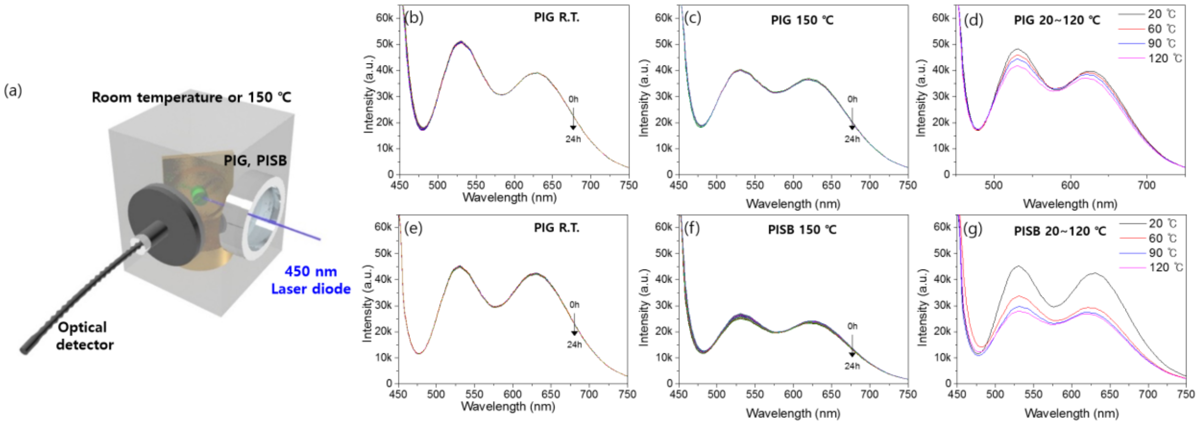

Figure S10. (a) Illustration of the optical measurement system used for the photosensitivity test. The PL spectra of the PIG plate at (b) room temperature, (c) 150 °C for 24 hrs, and (d) in a temperature range of 20 – 120 °C. Correspondingly, the PL spectra of the PISB plate at (e) room temperature, (f) 150 °C for 24 hrs, and (g) in a temperature range of 20 – 120 °C is shown.

To confirm the photosensitivity of the PIG sample at 150 °C, we designed a measurement system using a 450-nm laser diode and a thermal controllable holder. First, the power density of the blue laser diode is adjusted to the same value of the blue COB diode operated at the rated current (~450 W/cm^2). The PIG sample is then placed on the room-temperature or 150 °C sample holder and measured to obtain the spectra while under exposure to the laser diode for 24 hrs. The photosensitivity test of the PISB film was carried out to compare the results with those of the PIG plate test. During the 24-hr measurement at room temperature, the PL intensities both the PIG and PISB samples changed only slightly compared to the measurement results at 0 hrs. The PL intensities decreased as the applied temperature (20 ~ 150 °C) was increased over time due to thermal quenching of the phosphor. After reaching 150 °C, the PL measurement was performed, lasting 24 hrs. The results of the PIG plate at 24 hrs show that the intensity decreased by 2% compared to the PL intensity at 0 hrs. The results of the PISB film at 24 hrs show that the intensity decreased by 8% compared to the PL intensity at 0 hrs. We concluded that because the thermal conductivity of the PISB film is lower than that of the PIG plate, thermal degradation is apparently continuous.
